# Potential Way to Develop Dengue Virus Detection in *Aedes* Larvae as an Alternative for Dengue Active Surveillance: A Literature Review

**DOI:** 10.3390/tropicalmed9030060

**Published:** 2024-03-11

**Authors:** Yenny Rachmawati, Savira Ekawardhani, Nisa Fauziah, Lia Faridah, Kozo Watanabe

**Affiliations:** 1 Biomedical Science Master Program, Faculty of Medicine, Universitas Padjadjaran, Bandung 54211, Indonesia; yenny22001@mail.unpad.ac.id; 2 Faculty of Medicine, IPB University, Bogor 16680, Indonesia; 3 Department of Biomedical Science, Parasitology Division, Faculty of Medicine, Universitas Padjadjaran, Bandung 54211, Indonesia; savira@unpad.ac.id (S.E.); nisa@unpad.ac.id (N.F.); 4 Center for Marine Environmental Studies (CMES), Ehime University, Matsuyama, Ehime 790-8577, Japan

**Keywords:** dengue virus, *Aedes* larvae, dengue surveillance

## Abstract

The burden of dengue has emerged as a serious public health issue due to its impact on morbidity, mortality, and economic burden. Existing surveillance systems are inadequate to provide the necessary data for the prompt and efficient control of dengue. Passive surveillance of dengue cases may lead to underreporting and delayed mitigation responses. Improved dengue control program requires sensitive and proactive methods for early detection of dengue. We collected and reviewed existing research articles worldwide on detecting dengue virus in *Aedes* species larvae. Searches were conducted in PUBMED and Google Scholar, including all the studies published in English and Bahasa Indonesia. Twenty-nine studies were included in this review in terms of assay used, positivity rate, and dengue serotype detected. The presence of dengue virus in immature mosquitoes was mostly detected using reverse transcription PCR (RT-PCR) in pooled larvae. In one study, dengue virus was detected in larvae from laboratory-infected mosquitoes using enzyme-linked immunosorbent assay (ELISA). The positivity rate of dengue virus detection ranged from 0 to 50% in field-caught larvae. Although various methods can detect the dengue virus, further research encourages the use of low-cost and less laborious methods for active surveillance of dengue in larvae.

## 1. Introduction

Dengue is one of the most widespread and rapidly spreading vector-borne infections globally, especially in tropical and sub-tropical countries. Dengue fever is an acute systemic viral infection caused by dengue virus (DENV), which is transmitted through mosquito bites, mainly by *Aedes Aegypti* and *Aedes Albopictus*. Dengue has been endemic in 128 nations and is predicted to cause about 390 million infections every year [1,2,3]. Dengue has caused an economic and disease burden in endemic countries, where 70% of endemic regions are in Asia. In Southeast Asia, it is estimated that 2.9 million dengue cases occur annually, with 5906 deaths [2]. Despite the efforts of dengue control programs, the burden of dengue still becomes a problem for several tropical countries, including Indonesia. Moreover, the COVID-19 pandemic that affected people worldwide has masked the increase i dengue incidence and poses new challenges in dengue control, especially in dengue-endemic regions with highly transmitted COVID-19 infections and limited resources [4,5,6]. Several factors can be related to dengue outbreaks, including demography, environment, and social and ecological factors [7]. Advanced global development, including modernization of transportation, has contributed to the increased spread of dengue from endemic to non-endemic regions [7].

In recent times, there has been a shift in the clinical profile of dengue cases. Previously, the majority of cases affected children, but now more adults are being affected [8]. Adults tend to have non-specific symptoms and are at a higher risk of severe outcomes due to pre-existing health conditions and decreased organ function [9]. Early diagnosis is crucial for effective disease management to manage dengue effectively and to reduce the risk of dengue-related deaths and disease severity. Reducing dengue cases can be achieved by improving the prediction of outbreaks and detection methods through ongoing monitoring of both cases and the distribution of disease-carrying vectors. Obtaining more precise assessments of the dengue burden is vital to evaluate the effectiveness of prevention efforts. Enhanced surveillance systems and dedicated research are indispensable components of dengue prevention and control strategies [10]. Furthermore, at present, dengue vaccine is still not efficient enough, meaning that the prevention and control of dengue virus transmission still relies on depressing the mosquito population through several vector control programs, including active and continuous surveillance of cases and vectors [11]. The information provided by current vector monitoring on mosquito and larvae abundance is limited in terms of suitable sampling techniques, sample collecting locations, and sample collection times. Meanwhile, recent studies on detecting viruses in immature vectors have been primarily focused on establishing the phenomenon of vertical transmission. Despite using various methods, certain studies were unable to detect the DENV virus in immature mosquitoes [12,13]. However, the potential of this approach as a surveillance tool is yet to be studied in detail. This presents an opportunity to explore the feasibility of using this method for conducting disease surveillance. The current review aimed to summarize and discuss findings of dengue virus detection in larvae and provides important insights into the current state of knowledge on dengue virus detection in larvae, which will aid in the development of effective control strategies for this vector-borne disease.

## 2. Materials and Methods

### 2.1. Literature Search

Electronic searches were conducted in the PubMed and Google Scholar databases using keywords defined by the PICO method to identify any methods used to detect DENV infection in *Aedes* larvae (Table 1), including “Dengue” OR “DENV” AND “detect”, AND “larvae” OR “immature” to identify relevant articles published up until 15 August 2023. Searches included these words as well as truncated terms. A basic search was performed to identify commonly used terms in the literature describing dengue or DENV, surveillance, and larvae. We determined the most comprehensive literature search field combination. After duplicates were removed, articles were further screened based on titles and abstracts, and a thorough assessment of relevance was conducted by full text reading. Additional references were added if they were relevant to the topic and helpful for the discussion.

### 2.2. Selection Criteria

All full-text articles works were assessed for eligibility. The inclusion criteria used in the current review were articles indexed in PubMed or Google Scholar that detected dengue virus in immature mosquitoes. The authors excluded studies written in languages other than English or Bahasa Indonesia, review articles, abstract only articles, and gray articles.

### 2.3. Data Extraction

Data were extracted using a data extraction form and collected in Microsoft Excel. Data retrieved from various articles included authors, year of publication, study location, stage of mosquito collected, stage of mosquito when the assay was performed, method used, positivity rate, minimum infection rate, and DENV serotype detected. When the relevant data were unavailable or could not be obtained from the article, they were written as “not available” (N/A).

## 3. Results

### 3.1. Overview

**Figure 1:**
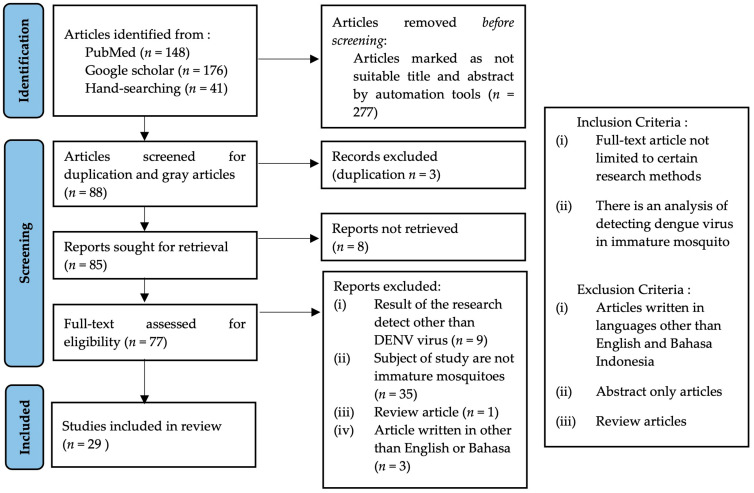
Flow diagram of identification, screening, and inclusion of studied included in this review.

The current review included 29 articles from 365 articles found in various online databases, also retrieved by hand-searching methods. As many as 88 articles passed the first screening and were then screened for duplicates. Eighty-five articles were sought for retrieval and seventy-seven articles remained for full-text review. A total of 48 articles were excluded for detecting viruses other than DENV (9 articles), detecting the virus in reared mosquitoes (35 articles), a review article (1 article), and for being written in languages other than English or Bahasa (3 articles), leaving only 29 studies considered relevant to be included in this review (Table S1). The flow of the literature search strategy is described in Figure 1.

### 3.2. Mosquito Stage and Population

**Figure 2:**
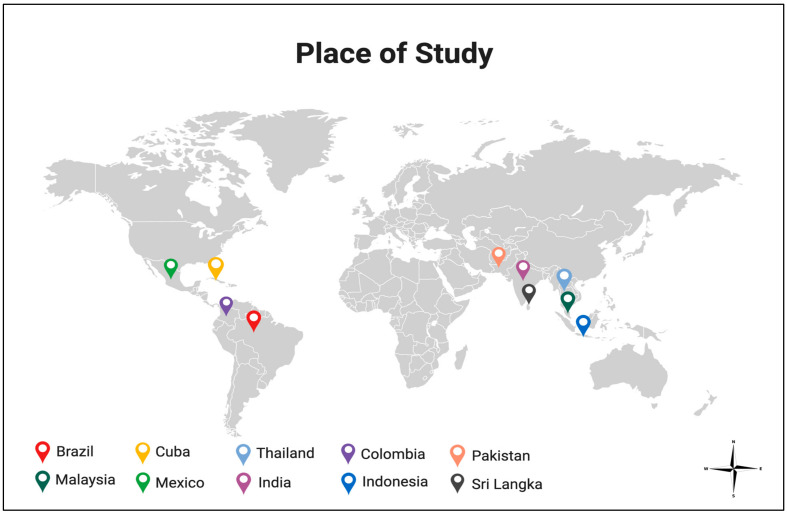
Map showing the geographical locations of the studies conducted in endemic countries: Brazil (12 studies), Malaysia (5 studies), Cuba (3 studies), Mexico (2 studies), Thailand (2 studies), India (1 study), Colombia (1 study), Indonesia (1 study), Pakistan (1 study), and Sri Lanka (1 study). Adapted from “World Map” by Biorender.com. Retrieved from https://app/biorender.com/biorender-templates. (accessed on 18 February 2024).

Most studies conducted that detect dengue virus in larvae aimed to investigate evidence of vertically transmitted DENV in mosquitoes. Fourteen of the twenty-nine (*n* = 14/29) articles in this review detect dengue virus in immature mosquitoes (larvae and pupae), while the rest of the studies detect the virus in both adult and immature mosquitoes. *Aedes Aegypti* was examined in 26 studies (Table 2), and *Aedes Albopictus* was examined in 14 studies (Table 3). Only eleven studies limited the age of the larvae to the 3rd and 4th instar, whilst others included all immature forms of mosquitoes. The largest proportion of the studies was conducted in Brazil (11/29). However, all studies identified were conducted in endemic countries, as shown in Figure 2. Almost all articles examined field-caught larvae (*n* = 26/29), while larvae from laboratory-infected mosquitoes were used in three articles. DENV detection was demonstrated in all three studies when testing larvae derived from laboratory-infected mosquitoes for virus detection. DENV also could be detected from filed-caught larvae in most studies (22/26) using various methods [14,15,16,17,18,19,20,21,22,23,24,25,26,27,28,29,30,31,32,33,34,35,36,37,38]. Twenty-six studies used pooled immature specimens when detecting dengue virus. 

### 3.3. Virus Detection Assay

Various methods were used to detect dengue virus in larvae and mosquitoes (Table 2). From the 29 studies included in the current review, the majority of studies used RT-PCR to detect DENV in immature mosquitoes either directly from larvae or inoculated into cell culture (*n* = 23/29). Other methods were the immunofluorescence technique (IFA) (*n* = 1/29), immunohistochemistry (PAP) (*n* = 1/29), and enzyme link immunosorbent assay (ELISA) (*n* = 1). Two of the twenty-nine (*n* = 2/29) articles compared IFA and PCR to detect dengue virus, and another study (*n* = 1/29) compared PAP and PCR. Dengue virus was detected in most studies (25/29), while four of the twenty-nine (*n* = 4/29) studies failed to detect the virus from larvae using PCR (*n* = 3/29) and IFA (*n* = 1) [13,39,40,41]. The positivity rate of DENV detection in larvae in the current review varies. The highest positivity rate of DENV detection in larvae hatched from laboratory-infected parents using PCR was 97.3%. 

## 4. Discussion

### 4.1. Larvae Surveillance

The World Health Organization (WHO) and every nation have agreed that dengue control requires strong multi-sectoral collaboration, consisting of vector control, ongoing surveillance, vaccine development, government commitment, public awareness and social mobilization, healthcare capacity development, and continuous research and innovation [9]. Effective case surveillance is important to detect ongoing dengue outbreaks in order to take prompt and rapid action and evaluate the progress of preventive efforts. Passive surveillance systems could result in underreporting, and multi-sectoral collaboration should be emphasized more in proactive surveillance, including case surveillance, vector surveillance, as well as environmental surveillance to provide more time to prevent small clusters from becoming large-scale outbreaks [42].

Vector surveillance through vector monitoring is crucial to provide information about distribution, density, larval habitat, and spatiotemporal risk factors related to dengue transmission as well as insecticidal susceptibility, which are important entomological information to execute vector control based on a prioritized area and season [43,44]. There are several vector monitoring methods, such as egg survey, larvae survey, and mosquito survey, to determine the density and risk of disease and provide the basis for evaluating the vector control effect [39]. Many different methods can be conducted to gather information about mosquito density, such as adult mosquito collection by human landing catch, human-baited double net trap, animal-baited trap net, adult mosquito collection using a light trap, morning resting adult mosquito collection, larvae collection by larvae pipette method, and larvae collection using ovitrap [45,46].

Even though a vector control program has been implemented by the Indonesian government, it has not been conducted in all areas. Moreover, the COVID-19 pandemic has generated a gap between expectation and reality in dengue vector control. A study stated that there is an increase in mosquito densities in the surrounding area due to a decrease or even halt in dengue vector control movement during the pandemic [43]. As dengue cases still existed and were still high during the pandemic, there is a driving pressure to create innovation in dengue control, including vector control through active vector surveillance.

In Indonesia, vector surveillance is done based on conventional larvae survey. Vector control is conducted through community empowerment in vector monitoring by Jumantik program. Jumantik or Juru Pemantau Jentik, is a squad monitoring larvae’s existence by entering the society’s house. Data gathered by Jumantik cadre is collected and reported to the health center to calculate the larvae indices, including house index (HI), breteau index (BI), and container index (CI), which will be used as consideration in making vector control policy [43]. HI is defined as percentage of houses infested with larvae or pupa, BI is number of positive containers for larvae or pupae in inspected houses, CI is percentage of water-holding containers invested with larvae or pupa [45]. HI, BI, CI and larvae-free indexes were then used as indicators for dengue transmission risk [44]. Stegomyia indices used to assess dengue transmission risk are as shown in Table 4 [45]. 

According to Wijayanti et al., HI, BI, and CI gathered from conventional larvae surveys have been inadequate for estimating dengue transmission risk considering that it might not represent the actual adult mosquito population and because larvae density was not always in accordance with the number of DENV cases [47]. This limitation was due to the larva’s ability to escape rapidly from sight and its capacity to remain submerged for a long period [48]. Moreover, vector surveillance is usually conducted for a short period, and sampling techniques have not been standardized among surveyed areas, so the data collected varied and could generate biases [44,49]. Several studies comparing the correlation between mosquito surveys and larvae surveys with dengue incidence showed that mosquito surveys could provide more accurate data to predict impending dengue outbreaks, both spatially and temporally [45,50]. However, tracing and tracking adult mosquitoes was not practical and required some skill to capture adult mosquitoes because they frequently fly to unreachable landing sites compared to larvae phases that are incapable of abandoning the site. Larvae sampling is easier, safer, and cheaper than sampling adult mosquitoes; therefore, immature sampling is the most effective method [51,52,53].

An alternative surveillance system that provides long-term vector surveillance and has good sensitivity is surveillance using an ovitrap. An ovitrap is an effective, inexpensive, and easy-to-use tool for improving dengue control and preventive programs. Ovitraps are defined as a simple container used to collect mosquito eggs. An ovitrap is preferable because it can detect eggs laid by both gravid mosquitoes and immature mosquitoes [44]. A study from Italy estimated that every five eggs collected in an ovitrap were proportionate to one person being bitten by a female *Aedes* [54]. An ovitrap could give more information about mosquito density values than a conventional larvae survey due to the preferential oviposition site of a gravid female mosquito [55]. An ovitrap is an active surveillance method to detect not only eggs laid by gravid mosquitoes but also immature mosquitoes and could provide entomological data in order to execute environmental clean-up and reduce mosquito breeding sites [44].

A surveillance system effectivity using ovitraps as a dengue vector control method has been proved in several countries. In Malaysia, surveillance using ovitraps in identified dengue hotspot areas shows a positive correlation between the ovitrap index (OI) and dengue cases [56,57,58]. Vector surveillance using an ovitrap followed by early detection and environmental awareness has been proved to help improve mosquito-borne disease transmission in Taiwan [59,60]. Twelve years of observational data about dengue occurrence and vector population using ovitraps in Brazil could obtain spatial patterns indicating the presence of persistent breeding sites, which has potential implications for vector control [61]. Moreover, ovitrap surveys allowed for the characterization of patterns of seasonal and spatial distribution of mosquito infestation, which showed that in addition to urbanization, rainfall was another factor that significantly increased the number of eggs and, therefore, resulted in a higher risk of dengue transmission [62]. Another study in Brazil also indicated that data gathered by ovitraps could be used for dengue incidence warning signals up to two months earlier and had a good predictive power for upcoming dengue outbreaks [48]. This finding was in accordance with a study carried out in Sri Lanka, which examined several area with repeated dengue epidemics, showing that mosquito density depicted in the OI is positively correlated with dengue incidence [63].

### 4.2. Detecting Dengue Virus in Larvae

Dengue cases are not affected by mosquito abundance but are closely related to the existence of DENV-infected mosquitoes [64]. Selvarajoo’s and Liew et al. demonstrated that DENV-infected mosquitoes can be detected one week prior to reported dengue cases. This significant positive correlation shows that detection of dengue positive mosquitoes can be used as an indicator of dengue transmission [64,65]. Moreover, a study conducted in Malaysia by Tan et al. found that there are asymptomatic dengue cases around detected dengue-positive mosquitoes and around suspected or confirmed dengue patients [66]. This indicates that a reactive vector control driven by dengue cases surveillance or after dengue cases are reported, as in the current reactive dengue control program, is already too late and might not describe actual cases [67]. These asymptomatic individuals could play a role as virus reservoirs and become a source of dengue infection, hence enhancing the persistence of dengue transmission in the absence of an epidemic [66]. Detection and characterization of local virus circulation in mosquito populations could better inform transmission risk assessment. 

Experimental studies have shown that several mosquito-borne flavivirus pathogens can be transmitted vertically in their insect vectors. The transovarial transmission of dengue virus has been observed in studies carried out, with DENV being detected in adult male specimens, those that do not feed on blood, as well as in immature stages or laboratory-reared adult mosquitoes. Rohani et al. demonstrate that in addition to horizontal virus transmission through DENV-infected mosquito bites to humans, there is vertical transmission in *Aedes* sp. reared under laboratory conditions until the fifth generation [68]. Vertical transmission could have important epidemiological consequences for dengue transmission as it can be a successful factor that maintains dengue virus persistence in nature by providing a temporary reservoir for the virus, because mosquito eggs are capable of surviving an unfavorable environment for long periods, even for more than a year [21,69].

Detection of the dengue virus in immature stages has been well documented. PCR showed a positivity rate of up to 97% from larvae progeny 21 days after mosquito infection. Other methods, such as IFA, could detect up to 68,6% of the same pools [31]. In nature, an increase in the dengue-positive immature mosquitoes may indicate the presence of infected parent mosquitoes [70]. Two studies conducted in India and Mexico showed different results, as they failed to detect the dengue virus in field-caught larvae using RT-PCR, even though DENV virus was detected in field-caught adult mosquitoes and reared mosquitoes from the same area [39,40]. This could be due to the small sample included in the study or might suggest that the vertical transmission of the DENV virus occurs at a very low rate. On the other hand, the positivity rate of DENV detection in larvae could reach as high as 50% from field-caught larvae using the same method [34]. The cause of the inadequate detection of dengue viruses in both larvae and adult mosquito samples can be attributed to a range of factors that go beyond vertical transmission. These factors include the various methodologies employed for larval and mosquito sampling and testing, which can significantly affect the accuracy of the results. The DENV virus was isolated using various RNA extraction techniques, although no method demonstrated superiority over the others. Studies that detected DENV in immature mosquitoes and those that could not do so used RNA extraction techniques, utilizing Trizol or commercial RNA extraction kits.

The method of pooling or individual sampling has the same impact on virus detection. When individual samples are collected, it significantly reduces the possibility of errors caused by introducing a misidentified sample into a large pool. This phenomenon might explain why three studies that examined pooled larvae consisting of 20 to 30 larvae could not detect DENV from pool larvae [39,40,41]. In contrast, pooling samples up to 50 larvae per pool is expected to increase the virus yield, making it more efficient to detect the virus. However, it has been found that the DENV virus can be detected using RT-PCR even from individual larvae despite the pooling method [26,34,35]. According to Pessanha et al., there seems to be equal sensitivity in detecting the DENV virus in individual samples and pools of larvae [34]. It also seems that the sensitivity was not affected by limiting the observation to only the 3rd and 4th instar stages [13,14,17,19,20,22,24,28,31,33,41].

PCR is considered superior to other method for the accuracy, sensitivity, and speed. A study in Mexico showed that PCR has a higher positivity rate than IFA when detecting the dengue virus from pooled larva, at 26% and 19%, respectively, in progeny from 7 days after dengue infection, to 97% and 68,6%, respectively, in progeny from 21 days after infection [31]. The increase in positivity rate associated with the later time of oviposition following the infection could be due to inadequate virus distribution to reproductive tissues before the initial batch of eggs is produced and laid. These findings were consistent with earlier laboratory reports that demonstrated the detection of the chikungunya virus (CHIKV) in *Aedes Aegypti* larvae obtained after 13–14 days post-infection by infectious blood meal. This confirmed that efficient dissemination of the virus within the mosquito to various secondary organs, presumably ovaries, was achieved on day 14 post-infection [71]. In order to detect DENV, most studies utilized nested reverse transcriptase PCR (RT-PCR) with DENV consensus primers, as outlined by Lanciotti [72]. While some studies also employed NS3 gene primers in laboratory-infected larvae, another study was unable to detect DENV in field-caught larvae [31,40]. To determine the DENV serotype, most studies employed a range of forward and reverse primers, including D1, D2, TS1, TS2, TS3, and TS4. All serotypes could be detected from immature mosquitoes and showed circulating DENV virus serotypes in the region. Pessanha even reported the coinfection of immature mosquitoes by two serotypes by detecting different serotypes of the dengue virus from individual larvae [34]. Even though PCR-based assays are a highly sensitive tool for detecting viruses, these assays are susceptible to contamination and the amplification of viral sequences that are present in the mosquito genome. Therefore, it is crucial to carefully evaluate the data obtained from PCR-based assays and consider all possible sources of experimental error before drawing any definitive conclusions.

The current method of DENV detection in mosquitoes usually includes the isolation and propagation of viruses, ELISA, or RT-PCR, which require highly specialized equipment, as well as highly trained personnel. Alternatively, Abraham et al., demonstrated that commercial NS1 antigen-based kits could be used to detect the DENV-NS1 antigen in mosquito pools with high sensitivity, as low as 2.5 pg DENV NS1 antigen [73,74]. Sylvestre et al., who compared a commercial NS1 antigen kit with qRT-PCR to detect DENV-2 in dried mosquitoes, demonstrated that NS1 has a higher sensitivity than PCR [75]. This finding will make surveillance of DENV prevalence in natural mosquito populations more affordable and feasible to be conducted in all endemic areas, especially developing regions of the world. Early detection of dengue positive mosquitoes could bring rapid and targeted prevention and mitigation of dengue epidemic risks. A previous laboratory study in Thailand demonstrated an inexpensive, rapid, ELISA-based screening technique to detect dengue in individual or pooled mosquitoes, either immature or adult. The percentage of positive pools ranged from 52% for DENV 2 to 83% for DENV 4 [28]. 

Detection of viruses in immature mosquitoes is also proven for vector-borne diseases other than dengue. As in dengue, some mosquito-borne viruses can be transmitted from female mosquitoes to their progeny, as evident in the presence of virus in non-bloodmeal male mosquitoes, and in immature mosquitoes of any sex [76]. A study by Tingstorm et al. found the Sindbis virus (SINV) in immature *Ochlerotatus* sp. mosquito gathered from a previous SINV outbreak area [77]. SINV RNA is still detected in mosquito larvae even 1 year after the outbreak, suggesting that the virus may hibernate in mosquito eggs and amplify during development [77]. This phenomenon explained an unexpected increase in the virus after rainy periods and led to an outbreak [76]. In a study carried out in Brazil, using RT-PCR on immature *Aedes* spp., the Zika virus (ZIKV) was found in immature stages at least five months prior to the peak of ZIKV-associated cases in the study region [78]. In line with Lee and Rohani, transovarial transmission of the dengue virus occurred between 7 to 14 days prior to the reporting of human cases [17]. According to Sambado et al., the pathogen can also be detected in an immature form in vectors other than mosquitoes. The study shows that *Borellia miyamotoi*, bacteria that cause tickborne relapsing fever, can be found in juvenile stages of *Ixodes pacificus* using nested-PCR and that all of the vector’s life stages carry the pathogen and pose some level of disease risk to humans [79]. A method to detect the virus or pathogen in the immature form of vectors other than by using PCR is demonstrated by Rosen et al. This study found that the Japanese encephalitis virus was detected in the larval stage of *Culex Tritaeniorhynchus* using the IFA method. An interesting finding is that viral detection was lower when measured in adults as compared with larvae from the same progenitor [80]. This was in line with the study conducted in Argentina that showed a diminishing infection rate observed between larvae and adult stages of *Culex* spp. for St. Louis encephalitis virus (SLEV). This indicates that there may be viral inactivation or alteration occurring during vector metamorphosis or when adults emerge [81].

The level of DENV positive larvae describes the level of vertical transmission in a specific location, which has epidemiological implications in terms of viral maintenance in vectors. A mathematical model predicts that vertical infection rates require at least 20% of DENV-positive larvae to significantly impact the dengue epidemic [69]. These phenomena indicated that larvae surveillance from the natural environment holds the potential to identify dengue transmission, and dengue-positive larvae abundance could provide an early warning signal of an impending dengue epidemic so that control intervention can be implemented to prevent the larger-scale outbreak [70]. Xenomonitoring in the larvae of *Aedes* spp. is not used as a routine tool in dengue control programs. The role of the vertical transmission rate is often ignored. A study by Murillo et al. demonstrated that a dengue outbreak is more difficult to control and more expensive when it is dominated by the strain with vertical transmission [82]. In such cases, traditional methods that target the elimination of adult mosquitoes may not be effective in controlling the outbreaks. As an alternative strategy, monitoring the infection rates in immature mosquitoes to eliminate the larvae infected with the virus transmitted through vertical transmission can be implemented to control dengue outbreaks. A study in Sri Lanka showed a similarity between the DENV serotypes observed in patients and those in immature *Aedes* spp. larvae pools collected from the patients’ daily environments [22]. This finding provides potential evidence that mosquitoes hatching from infected eggs could contribute to dengue outbreaks in the region, and surveillance of immature forms of mosquitoes can be as informative as monitoring adults for detecting the DENV serotype circulating in the mosquito population. This suggest that monitoring mosquito larvae may provide a valuable insights into the transmission dynamics of DENV. Xenomonitoring of a single larvae even demonstrated that the immature mosquitoes can be coinfected by two serotypes via transovarial transmission [34]. It raises questions about the relationship between this phenomenon and the transmission of specific DENV serotypes in particular locations, as well as the clinical severity of cases. 

The RT-PCR technique that is currently used to detect the dengue virus in larvae is a powerful, highly sensitive, virus surveillance tool, yet it is an expensive and laborious technique. There is no study demonstrating the use of other methods of DENV detection in immature mosquitoes that are more affordable and less laborious, such as commercial NS1 antigen-based kits. Further research may explore the potential of rapid diagnostic test (RDT) kits for detecting dengue virus in *Aedes* larvae. If effectively proven, the early detection of DENV in the immature mosquito population would afford greater efficiency of vector control activities in those dengue-endemic areas.

The collected data in this review have limitations due to their heterogeneity in assay methods, pool sizes, larval stages, and the unavailability of certain data, which negatively affect statistical analysis. A limitation of this paper is that the data used come from an endemic region and cannot describe the potential of testing methods in non-endemic regions.

## 5. Conclusions

Integrated dengue surveillance should ensure that increased dengue transmission is detected early, and that the response is rapid and appropriate. Vector control as an anticipation of an outbreak was much more effective than control efforts that were implemented after disease transmission had begun. A proactive strategy is important for the dengue vector surveillance program. Immature sampling is more efficient than the adult-catching method. Viral surveillance would be inefficient if confirmation of DENV were not rapid and not practical to use by public health workers with minimum training. The dengue virus could be detected from larvae vectors using some methods, such as PCR and ELISA. Larvae surveillance has the potential to identify dengue transmission during epidemic and inter-epidemic periods, and dengue positive-larvae detection could provide an early warning signal of an impending dengue epidemic. Detecting dengue viral antigens using more affordable and less laborious techniques, such as antigen detection kits in larvae vectors, would benefit dengue control programs. Further research is needed to determine the true epidemiological significance of the transovarial transmission rate. Large-scale, multicenter studies, including endemic and non-endemic regions, are needed to further evaluate the potential role of larval surveillance and detection and to identify to what extent the detection of DENV in larvae is correlated to dengue incidence and whether it can serve as active surveillance. 

## Figures and Tables

**Table 1:** The PICO method to define keywords for the searching strategy.

Population	*Aedes* Sp. Larvae
Intervention/Exposure	Any methods used for virus detection, such as PCR, ELISA, or immunofluorescence assay
Control	-
Outcome	DENV infection

**Table 2:** Summary of studies detecting dengue virus in *Aedes Aegypti* larvae.

No	Author (Year)	Place of Study	Method of Assay	Positivity Rate (Positive Pools/Total Pools ×100)	DENV	Source of Infection	Pool/Individual	Larval Age	Ref.
1	Watts DM., et al. (1985)	Thailand	DFA	0	NA	Nature	Pool of 25 or less	3rd and 4th instar	[13]
2	Castro MG. et al. (2004)	Brazil	Nested RT-PCR from cell culture	32.4% (*n* = 12)	2	Lab	4 to 23/pool	4th instar	[14]
3	Medeiros AS., et al. (2018)	Brazil	Nested RT-PCR	8.7% (*n =* 4)	1, 2, 4	Nature	Pool of 40 or less	NA	[15]
4	Khan J., et al. (2017)	Pakistan	Nested RT-PCR	20% (*n* = 2)	2, 3	Nature	30/pool	NA	[16]
5	Lee HL., et al. (2005)	Malaysia	PAP staining from cell culture	2% (*n* = 3)	NA	Nature	25/pool	3rd instar	[17]
6	Teixeira AF., et al. (2021)	Brazil	qRT-PCR	13.3% (*n* = 4)	NA	Nature	15/pool	NA	[18]
7	Da Costa CF., et al.(2017)	Amazona (Brazil)	qRT-PCR	47.9% (*n* = 70)	1, 2, 4	Nature	Pool of 30 or less	3rd and 4th instar	[19]
8	Andrade, EHP., et al. (2022)	Brazil	qRT-PCR	32.1% (*n* = 9)	1, 2, 3, 4	Nature	Pool of 10 or less	3rd and 4th instar	[20]
9	Mulyatno KC., et al. (2012)	Indonesia	RT- PCR	10.7 (*n* = 3)	1, 2	Nature	20/pool	NA	[21]
10	Wijesinghe (2021)	Sri Lanka	RT-PCR	9.8% (*n* = 12)	1, 2, 3, 4	Nature	Pool of 6 to 70	3rd and 4th instar	[22]
11	Vilela AP., et al. (2006)	Brazil	RT-PCR	0.9% (*n* = 1)	3	Nature	Pool of 50 or less	NA	[23]
12	Cecilio SG., et al. (2015)	Brazil	RT-PCR	7.4% (*n* = 4)	NA	Nature	Pool of 40 or less	4th instar	[24]
13	Pinheiro VCS., et al. (2005)	Brazil	RT-PCR	11.86% (*n* = 7)	3	Nature	4 to 49/pool	NA	[25]
14	Teo CHJ, et al. (2017)	Malaysia	RT-PCR	25% (*n* = 4)	2, 3, 4	Nature	Individual	NA	[26]
15	Rohani A, et al. (2014)	Malaysia	RT-PCR	5 pools	2, 3	Nature	15 to 20/pool	NA	[27]
16	Sithiprasasna R., et al. (1994))	Thailand	ELISA	DEN 1 = 63% (*n* = 29); DEN 2 = 51% (*n* = 21); DEN 3 = 69% (*n* = 24); DEN 4 = 83% (*n* = 35)	1, 2, 3, 4	Lab	1 to 100/pool	4th instar	[28]
17	Gutierrez-Bugallo G., et al. (2018)	Cuba	RT-PCR	33.3% (*n* = 3)	3	Nature	30/pool	NA	[29]
18	Granados JSM., et al. (2022)	Colombia	RT-PCR	31.25% (*n* = 5)	1, 2, 3	Nature	20/pool	NA	[30]
19	Sanchez-Vargas I. et al. (2018)	Mexico	IFA and RT-N-PCR from cell culture	E2-7d PCR/IFA = 26%/19.3%; E2-10d PCR/IFA = 55%/55%; E2-21d PCR/IFA = 97.3%/68.6%	2	Lab	20/pool	4th instar	[31]
20	Gutierrez-Bugallo G., et al. (2017)	Cuba	RT-PCR	33.3% (*n* = 37)	1, 2, 3, 4	Nature	30 to 55/pool	NA	[32]
21	Rohani A., et al. (2007)	Malaysia	RT-PCR and PAP staining from cell culture	RT-PCR = 5% (*n* = 19); PAP staining from cell culture = 8.7% (*n* = 33)	1, 3	Nature	10/pool	3rd and 4th instar	[33]
22	Pessanha JEM. et al. (2011)	Brazil	RT-PCR	Individual = 37.5% (*n* = 110); pool = 37.3% (*n* = 53)	1, 2, 3	Nature	Individual and 2 to 10/pool	NA	[34]
23	Johari NA., et al. (2019)	Malaysia	Nested RT-PCR	2.47% (*n* = 9)	1, 2, 3, 4	Nature	Individual	NA	[35]
24	Sivan A., et al. (2016)	India	RT-PCR	0	3	Nature	20/pool	NA	[39]
25	Gunther J., et al. (2007)	Mexico	RT-PCR	0	2, 3, 4	Nature	20/pool	NA	[40]
26	Zeidler JD., et al. (2007)	Brazil	RT-PCR	0	NA	Nature	Pool of 30 or less	3rd and 4th instar	[41]

**Table 3:** Summary of studies detecting dengue virus in *Aedes* Albopictus larvae.

No	Author (Year)	Place of Study	Method of Assay	Positivity Rate (Positive Pools/Total Pools ×100)	DENV	Source of Infection	Pool/Individual	Larval Age	Ref.
1	Castro MG. et al. (2004)	Brazil	Nested RT-PCR from cell culture	46.2% (*n* = 18)	2	Lab	4 to 23/pool	4th instar	[14]
2	Medeiros AS., et al. (2018)	Brazil	Nested RT-PCR	0% (*n* = 0)	1, 2, 4	Nature	Pool of 40 or less	NA	[15]
3	Khan J., et al. (2017)	Pakistan	Nested RT-PCR	14.29% (*n* = 1)	2, 3	Nature	30/pool	NA	[16]
4	Lee HL., et al. (2005)	Malaysia	PAP staining from cell culture	0.9% (*n* = 7)	NA	Nature	25/pool	3rd instar	[17]
5	Wijesinghe (2021)	Sri Lanka	RT-PCR	8.1% (*n* = 4)	1, 2, 3, 4	Nature	Pool of 6 to 70	3rd and 4th instar	[22]
6	Teo CHJ, et al. (2017)	Malaysia	RT-PCR	25.7% (*n* = 73);	2, 3, 4	Nature	Individual	NA	[26]
7	Rohani A, et al. (2014)	Malaysia	RT-PCR	18 pools	2, 3,	Nature	15 to 20/pool	NA	[27]
8	Rohani A., et al. (2007)	Malaysia	RT-PCR and PAP staining from cell culture	RT-PCR = 1.1% (*n* = 6); PAP staining from cell culture = 3.1% (*n* = 17)	1, 3	Nature	10/pool	3rd and 4th instar	[33]
9	Pessanha JEM. et al. (2007)	Brazil	RT-PCR	Individual = 50% (*n* = 4); pool = 50% (*n* = 1)	1, 2, 3	Nature	Individual and 2 to 10/pool	NA	[34]
10	Johari NA., et al. (2019)	Malaysia	Nested RT-PCR	2.05% (*n* = 21)	1, 2, 3, 4	Nature	Individual	NA	[35]
11	Piedra LA., et al. (2022)	Cuba	RT-PCR	26.67% (*n* = 4)	3	Nature	30/pool	NA	[36]
12	De Figueiredo ML., et al. (2010)	Brazil	RT-PCR	11.5% (*n* = 3)	1, 2, 3	Nature	10/pool	NA	[37]
13	Serufo JC., et al. (1993)	Brazil	IFA AND PCR	(*n* = 2)	1	Nature	Pool of 30 or less	NA	[38]
14	Sivan A., et al. (2016)	India	RT-PCR	0	3	Nature	20/pool	NA	[39]

**Table 4:** Stegomyia indices of dengue transmission risk.

Larval Index	Dengue Transmission Risk
BI < 5	Low risk of dengue transmission
BI ≥ 5	Risk of transmission
BI ≥ 10	Risk of outbreak
BI ≥ 20	Risk of regional transmission

(BI: Breteau index).

## Data Availability

No new data were created.

## Supplementary Materials

The following supporting information can be downloaded at: https://www.mdpi.com/article/10.3390/tropicalmed9030060/s1, Table S1: Summary of the collected data of the included studies.
