# Supplementary material for: Potential Way to Develop Dengue Virus Detection in Aedes Larvae as an Alternative for Dengue Active Surveillance: A Literature Review
Source: Trop Med Infect Dis. 2024 Mar 11;9(3):60. doi: 10.3390/tropicalmed9030060 (PMC10975107; doi:10.3390/tropicalmed9030060)
Supplement: Supplementary file 1 [file tropicalmed-09-00060-s001.zip › tropicalmed-2856008-supplementary.pdf]

Tropical Medicine and Infectious Disease

MDPI

Supplementary Materials:

Table S1. Dengue Virus Detection in Larvae

No	Author (Year)	Place of study	Method of assay	Number of sample		Number of pools		Positivity rate (Positive pools/Total pools x100)		DENV	Source of infection	Pool/not (Larvae)	Larval Age	Ref
				Immature Aegypti	Immature Albopictus	Immature Aegypti	Immature Albopictus	Immature Aegypti	Immature Albopictus					
1	Watts DM., et al. (1985)	Thailand	DFA	5839	NA	260	NA	0	NA	NA	nature	pool of 25 or less	3rd and 4th instar	[13]
2	Castro MG. et al. (2004)	Brazil	nested RT-PCR from cell culture	521	284	37	39	32,4% (12)	46,2% (n=18)	DENV 2	Lab	4 to 23/pool	4th instar	[14]
3	Medeiros AS., et al. (2018)	Brazil	nested RT-PCR	1186	147	46	13	8,7% (4)	0% (n=0)	DENV 1, DENV 2, DENV 4	nature	pool of 40 or less	NA	[15]
4	Khan J., et al (2017)	Pakistan	nested RT-PCR	300	210	10	7	20% (n=2)	14,29% (n=1)	DENV 2, DENV 3	nature	30/pool	NA	[16]
5	Lee HL., et al. (2005)	Malaysia	PAP staining from cell culture	3579	19343	150	777	2% (n=3)	0,9% (n=7)	NA	nature	25/pool	3rd instar	[17]
6	Teixeira AF., et al. (2021)	Brazil	qRT-PCR	450	NA	30	NA	13,3% (n=4)	NA	NA	nature	15/pool	NA	[18]
7	Da Costa CF., et al.(2017)	Amazon	qRT-PCR	3956	NA	146	NA	47,9% (n=70)	NA	DENV 1, DENV 2, DENV 4	nature	pool of 30 or less	3rd and 4th instar	[19]
8	Andrade, EHP., et al. (2022)	Brazil	qRT-PCR	NA	NA	28	NA	32,1% (n=9)	NA	DENV 1, DENV 2, DENV 3, DENV 4	nature	pool of 10 or less	3rd and 4th instar	[20]
9	Mulyatno KC., et al. (2012)	Indonesia	RT- PCR	550	NA	28	NA	10,7 (n=3)	NA	DENV 1, DENV 2	nature	20/pool	NA	[21]
10	Wijesinghe (2021)	Sri Lanka	RT-PCR	NA	NA	49	122	9,8% (n=12)	8,1% (n=4)	DENV 1, DENV 2, DENV 3, DENV 4	nature	pool of 10 or less	3rd and 4th instar	[22]

11	Vilela AP., et al. (2006)	Brazil	RT-PCR	5573	NA	101	NA	0,9% (1)	NA	DENV 3	nature	pool of 50 or less	NA	[23]
12	Cecilio SG., et al. (2015)	Brazil	RT-PCR	945	168	54 (not specified)		(n=4)	NA	NA	nature	pool of 40 or less	4th instar	[24]
13	Pinheiro VCS., et al.(2005)	Brazil	RT-PCR	1142	NA	59	NA	11,86% (n=7)	NA	DENV 3	nature	4 to 49/pool	NA	[25]
14	Teo CHJ, et al. (2017)	Malaysia	RT-PCR	16	284	16	284	25% (n=4)	25,7% (n=73) ;	DENV 2, DENV 3, DENV 4	nature	individual	NA	[26]
15	Rohani A, et al. (2014)	Malaysia	RT-PCR	137	2703	363 (not specified)		5 pools	18 pools	DENV 2, DENV 3,	nature	15 to 20/pool	NA	[27]
16	Sithiprasasna R., et al. (1994))	Thailand	ELISA	NA	NA	DEN 1 = 46; DEN 2 = 41; DEN 3 = 35; DEN 4 = 42	NA	DEN 1 = 63% (n=29); DEN 2 = 51% (n=21); DEN 3 = 69% (n=24) ; DEN 4 = 83% (n=35)	NA	DENV 1, DENV 2, DENV 3, DENV 4	Lab	1 to 100/pool	4th instar	[28]
17	Gutierrez-Bugallo G., et al. (2017)	Cuba	RT-PCR	270	NA	9	NA	33,3% (n=3)	NA	DENV 3	nature	30/pool	NA	[29]
18	Granados JSM., et al. (2022)	Colombia	RT-PCR	366	NA	16	NA	31,25% (n=5)	NA	DENV 1, DENV 2, DENV 3, CHIKV, ZIKV, YFV	nature	20/pool	NA	[30]
19	Sanchez-Vargas I. et al. (2018)	Mexico	IFA and RT-N-PCR from cell culture	E2-7d PCR/IFA= 2380/2380 ; E2-10d PCR/IFA = 1420/90; E2-21d PCR/IFA = 760/1020	NA	E2-7d PCR/IFA = 119/119 ; E2-10d PCR/IFA = 71/49; E2-21d PCR/IFA = 38/51	NA	E2-7d PCR/IFA = 26%/19,3% ; E2-10d PCR/IFA = 55%/55% ; E2-21d PCR/IFA = 97,3% 68,6%	NA	DENV 2	Lab	20/pool	4th instar	[31]
20	Gutierrez-Bugallo G., et al. (2018)	Cuba	RT-PCR	NA	542	NA	26	33,3% (n=37)	NA	DENV 1, DENV 2, DENV 3, DENV 4	nature	30 to 55/pool	NA	[32]

Tropical Medicine and Infectious Disease

MDPI

21	Rohani A., et al. (2007)	Malaysia	RT-PCR and PAP staining from cell culture	3780	5530	378	553	RT-PCR = 5% (n=19) ; PAP staing from cell culture = 8,7% (n=33)	RT-PCR = 1,1% (n=6) ; PAP staing from cell culture = 3,1% (n=17)	DENV1 , DENV 3	nature	10/pool	3rd and 4th instar	[33]
22	Pessanha JEM. et al. (2007)	Brazil	RT-PCR	1400	17	Individual = 293 ; pool = 142	Individual = 8; pool = 2	Individual = 37,5% (n=110) ; pool = 37,3% (n=53)	Individual = 50% (n=4) ; pool = 50% (n=1)	DENV 1, DENV 2, DENV 3	nature	individual and 2 to 10/pool	NA	[34]
23	Johari NA., et al. (2019)	Malaysia	nested RT-PCR	364	1025	364	1025	2,47% (n=9)	2,05% (n=21)	DENV 1, DENV 2, DENV 3, DENV 4	nature	Individual	NA	[35]
24	Piedra LA., et al. (2022)	Cuba	RT-PCR	NA	450	NA	15	NA	26,67% (n=4)	DENV 3	nature	30/pool	NA	[36]
25	De Figueiredo ML., et al. (2010)	Brazil	RT-PCR	270	NA	9	NA	NA	11,5% (n=3)	DENV 1, DENV 2, DENV 3	nature	10/pool	NA	[37]
26	Serufo JC., et al. (1993)	Brazil	IFA AND PCR	NA	1128 ;	NA	NA	NA	(n=2)	DENV 1	nature	pool of 30 or less	NA	[38]
27	Sivan A., et al. (2016)	India	RT-PCR	89	691	6	23	0	0	DENV 3	nature	20/pool	NA	[39]
28	Gunther J., et al. (2007)	Mexico	RT-PCR	620	NA	31	NA	0	NA	DENV 2, DENV 3, DENV 4	nature	20/pool	NA	[40]
29	Zeidler JD., et al. (2007)	Brazil	RT-PCR	1172	NA	44	NA	0	NA	NA	nature	pool of 30 or less	3rd and 4th instar	[41]
